# Evaluation of Mucosal and Systemic Immune Responses Elicited by GPI-0100- Adjuvanted Influenza Vaccine Delivered by Different Immunization Strategies

**DOI:** 10.1371/journal.pone.0069649

**Published:** 2013-07-31

**Authors:** Heng Liu, Harshad P. Patil, Jacqueline de Vries-Idema, Jan Wilschut, Anke Huckriede

**Affiliations:** Department of Medical Microbiology, Molecular Virology Section, University Medical Center Groningen, University of Groningen, Groningen, The Netherlands; The University of Adelaide, Australia

## Abstract

Vaccines for protection against respiratory infections should optimally induce a mucosal immune response in the respiratory tract in addition to a systemic immune response. However, current parenteral immunization modalities generally fail to induce mucosal immunity, while mucosal vaccine delivery often results in poor systemic immunity. In order to find an immunization strategy which satisfies the need for induction of both mucosal and systemic immunity, we compared local and systemic immune responses elicited by two mucosal immunizations, given either by the intranasal (IN) or the intrapulmonary (IPL) route, with responses elicited by a mucosal prime followed by a systemic boost immunization. The study was conducted in BALB/c mice and the vaccine formulation was an influenza subunit vaccine supplemented with GPI-0100, a saponin-derived adjuvant. While optimal mucosal antibody titers were obtained after two intrapulmonary vaccinations, optimal systemic antibody responses were achieved by intranasal prime followed by intramuscular boost. The latter strategy also resulted in the best T cell response, yet, it was ineffective in inducing nose or lung IgA. Successful induction of secretory IgA, IgG and T cell responses was only achieved with prime-boost strategies involving intrapulmonary immunization and was optimal when both immunizations were given via the intrapulmonary route. Our results underline that immunization via the lungs is particularly effective for priming as well as boosting of local and systemic immune responses.

## Introduction

The aim of mucosal immunization against respiratory virus infections is the induction of local immunity at the port of pathogen entry. In particular, mucosal antibodies can readily neutralize invading viruses at the luminal site of the epithelial layer and prevent their entry into host cells. Such an immune exclusion effect is mainly mediated by secretory IgA (SIgA), which is effectively induced by mucosal but not parenteral immunization [1–5]. Moreover, intracellular viruses can be neutralized during transcytosis of dimeric SIgA through the epithelial layer. Furthermore, for rapidly changing viruses like influenza virus, SIgA has been shown to be more cross-reactive than IgG and to neutralize antigen-drifted homosubtypic and even antigen-shifted heterosubtypic virus strains [6]. 

Despite the advantage of mucosal immunization for the induction of SIgA responses, the mucosal route is suboptimal for the induction of systemic antibody responses [7–9]. In case of influenza, systemic antibodies are important since they contribute to protection against virus replication in the lungs and are the only correlate of protection so far recognized by regulatory authorities [10]. Furthermore, due to the default Th2-oriented nature of mucosal immunity, mucosal immunization shows limited induction of Th1-related antibody subtypes (eg. IgG2a in Balb/c mice), which are preferable for viral clearance [11–15].

A potential way to combine the advantages of mucosal and systemic immunization involves prime-boost strategies with mucosal priming and systemic boosting or vice-versa. Several studies have investigated such strategies, but the majority of these make use of DNA vaccines and/or recombinant virus vaccines during priming, boosting or both [16–26]. So far, little is known about prime-boost strategies for optimization of mucosal and systemic immune responses to protein-based influenza vaccines. A study in horses using an ISCOM-adjuvanted influenza vaccine showed that intranasal boosting after intramuscular (IM) priming does not have much effect on serum IgG levels, but results in low and transient SIgA and IgG responses in nose washes [18]. However, no comparison was performed with alternative immunization strategies.

We earlier showed that GPI-0100, a semi-synthetic saponin-derivative, is a very effective adjuvant for influenza subunit vaccine administered via not only the intramuscular, but also the intranasal and particularly the intrapulmonary route [27,28]. Here, we used GPI-0100-adjuvanted influenza vaccine to identify an immunization strategy that effectively elicits influenza-specific immune responses at both mucosal and systemic sites. To this end, we compared the immune responses elicited by two mucosal strategies with the adjuvanted influenza vaccine to the responses obtained by a strategy involving a mucosal prime followed by a systemic booster immunization. Two different mucosal administration routes were evaluated: intranasal (IN) and intrapulmonary (IPL). We observed that systemic boosting was not as effective as mucosal boosting for induction of mucosal SIgA. Systemic boosting enhanced systemic IgG titers to higher levels than mucosal boosting in IN-primed, but not in IPL-primed mice. Yet, systemic boosting generally stimulated stronger Th1 cellular immunity than mucosal boosting. All the immunization strategies we tested in the current study provided complete protection against influenza virus infection.

## Materials and Methods

### GPI-0100

GPI-0100 was purchased from Hawaii Biotech, Inc. (Aiea, HI, USA) and was stored at 4 °C. A 10 mg/ml stock solution was prepared in HBS buffer (5 mM Hepes, 150 mM NaCl and 0.1 mM EDTA, pH 7.4) as described previously [27].

### Subunit vaccine and challenge virus preparation

A stock of A/Puerto Rico/8/34 (H1N1) influenza virus (PR8) propagated on Madin-Darby canine kidney (MDCK) cells was kindly provided by Solvay Biologicals (Weesp, Netherlands) and further propagated on embryonated eggs. Virus titer was determined by measuring the tissue culture infectious dose 50 (TCID_50_) [27].

For subunit vaccine preparation, the procedure was as previously described [27]. Briefly, PR8 virus was inactivated by β-propiolactone (0.1% in citrate buffer, freshly prepared). The inactivated virus sample was dialyzed against HBS buffer (5 mM Hepes, 150 mM NaCl and 0.1 mM EDTA, pH 7.4) and then solubilized by Tween 80 (0.6 mg/ml) and hexadecyltrimethylammonium bromide (CTAB, 3.0 mg/ml). The viral nucleocapsid was further removed from the preparation by ultracentrifugation. Subsequently, detergents were removed by Biobeads SM2 (634 mg/ml, Bio-Rad, Hercules, Canada) pre-washed with methanol.

Protein content of the subunit material was determined by a modified Lowry assay [29]. Hemagglutinin (HA) content was assumed to be equal to the total protein for subunit material based on sodium dodecyl sulfate polyacrylamide gel electrophoresis (SDS-PAGE) results which indicate presence of only minor amounts of other viral proteins [30]. Vaccines were mixed at the indicated amounts of subunit and GPI-0100 just before immunization. 

### Animal handling

The protocol for the animal experiments described here was approved by the Ethics Committee on Animal Research of the University of Groningen.

For immunization experiments, female Balb/c mice (Harlan, Horst, Netherlands) aged 8–10 weeks were grouped (n = 6 per group) and immunized IM, IN or IPL with 1 µg PR8 subunit vaccine with or without 15 µg GPI-0100 in a two-dose immunization regimen with a 20 day interval (Table 1). For IM immunizations, vaccines in 50 µl were divided over both hind legs. For IN immunizations, vaccines in 5 µl were slowly delivered with a pipet and divided over both nares thus confining the antigen to the nose [31]. For IPL immunizations, mice were brought to an upright position after isoflurane anesthesia and the trachea was intubated with a modified Autoguard catheter (Becton Dickinson BV, Breda, Netherlands). Vaccines in 50 µl were then delivered with the help of an IA-1C Microsprayer Aerosolizer attached to a FMJ-250 High Pressure Syringe (both from Penn-Century Inc., Wyndmoor, PA, USA).

**Table 1:** Immunization scheme.

Treatment Groups	Vaccine formulation	Administration route	
		Day 0	Day 20
2M	1 µg PR8 subunit	IM	IM
2N	1 µg PR8 subunit + 15 µg GPI-0100	IN	IN
N+M	1 µg PR8 subunit + 15 µg GPI-0100	IN	IM
2P	1 µg PR8 subunit + 15 µg GPI-0100	IPL	IPL
P+M	1 µg PR8 subunit + 15 µg GPI-0100	IPL	IM

Pre-boost blood samples were collected on day 20 by orbital puncture prior to the second immunization. For immunization experiments, mice were sacrificed on day 27 and nose wash, lung wash, blood and spleen samples were collected for ex vivo immuno-assays. Mucosal wash samples were collected in 1 ml phosphate-buffered saline (PBS) containing protease inhibitor (Complete Protease Inhibitor Cocktail, Roche, IN, USA).

For challenge experiments, mice received the immunization regimen as described above. Pre-boost and pre-challenge serum samples were collected on day 20 and day 34 by orbital puncture prior to immunization or virus infection, respectively. On day 34, mice were infected intranasally with 200 TCID_50_ PR8 influenza virus in 50 µl of HBS buffer. The virus infection was carried out under isoflurane anesthesia to ensure deposition of the virus into the lungs. Mice were monitored twice a day at fixed time points for clinical signs of illness including weight loss and changes in behavior or appearance. Mice were bled and sacrificed on day 37. Nasal wash, serum and spleen samples were collected for immuno-assays. The lung lobes were collected in 1 ml PBS for homogenization and the processed samples were stored at -80°C for later determination of lung virus titers.

### IgA, IgG, IgG1 and IgG2a ELISA

H1N1-specific antibody responses were determined by ELISA as previously described [28]. For nasal SIgA and IgG responses, OD 492 of individual samples is given. For lung SIgA and IgG responses, the average OD 492 (with the standard error of the means (S.E.M.)) for each group at each dilution was calculated. For serum IgG response, the titer for individual samples was calculated as the^10^ log of the reciprocal of the sample dilution corresponding to an OD 492 of 0.2. For calculation purposes, sera with titers below the detection limit were assigned an arbitrary titer corresponding to half of the detection limit.

Calibration plates for IgG1 and IgG2a assay were coated with 0.1 µg goat anti-mouse IgG (SouthernBiotech). Increasing concentrations of purified mouse IgG1 or IgG2a (SouthernBiotech) were added to the plates. IgG1 and IgG2a responses detected from individual sample are given as concentration (μg/ml) of H1N1-specific IgG1 and IgG2a.

### Hemagluttination inhibition (HAI)

Serum samples were processed and HAI titers were determined as described previously [28]. ^2^log HAI titers for individual mice are presented.

### ELISPOT

H1N1-specific IFN-γ and IL-4 responses were determined by ELISPOT assays as previously described [27]. Numbers of influenza-specific IFN-γ- or IL-4-secreting cells per 5x10^5^ splenocytes for each mouse are given.

### Virus titration

Lungs collected from the immunized and challenged mice were homogenized in PBS and stored at −80°C until use. Virus titers were determined by inoculating serial dilutions of the clarified homogenates on MDCK cells, as described earlier [28]. The titers of individual mice are given and the results are presented as^10^ log virus titer per gram of lung tissue.

### Statistics

The unpaired Student’s t-test was used to determine if the differences in influenza-specific responses observed between groups of mice were significant. A p value of p < 0.05 was considered significant. Spearman (nonparametric) correlation analysis was performed to assess the relationship observed between serum and mucosal influenza-specific IgG responses.

## Results

### Effect of immunization strategy on mucosal antibody titers

Since induction of a mucosal SIgA response is the central aim of mucosal immunization, we first evaluated the effect of the immunization strategy on the induced SIgA response. Mice received GPI-0100-adjuvanted influenza vaccine via the IN or IPL route on day 0 and received a second immunization on day 20 via the same mucosal route or IM. A control group immunized twice IM with non-adjuvanted vaccine served as a reference. Six mice of each group were sacrificed one week after the booster immunization for the collection of lung wash samples. The other six mice of each group were challenged with PR8 virus two weeks after the booster and sacrificed three days later for collection of nose wash samples.

**Figure 1:**
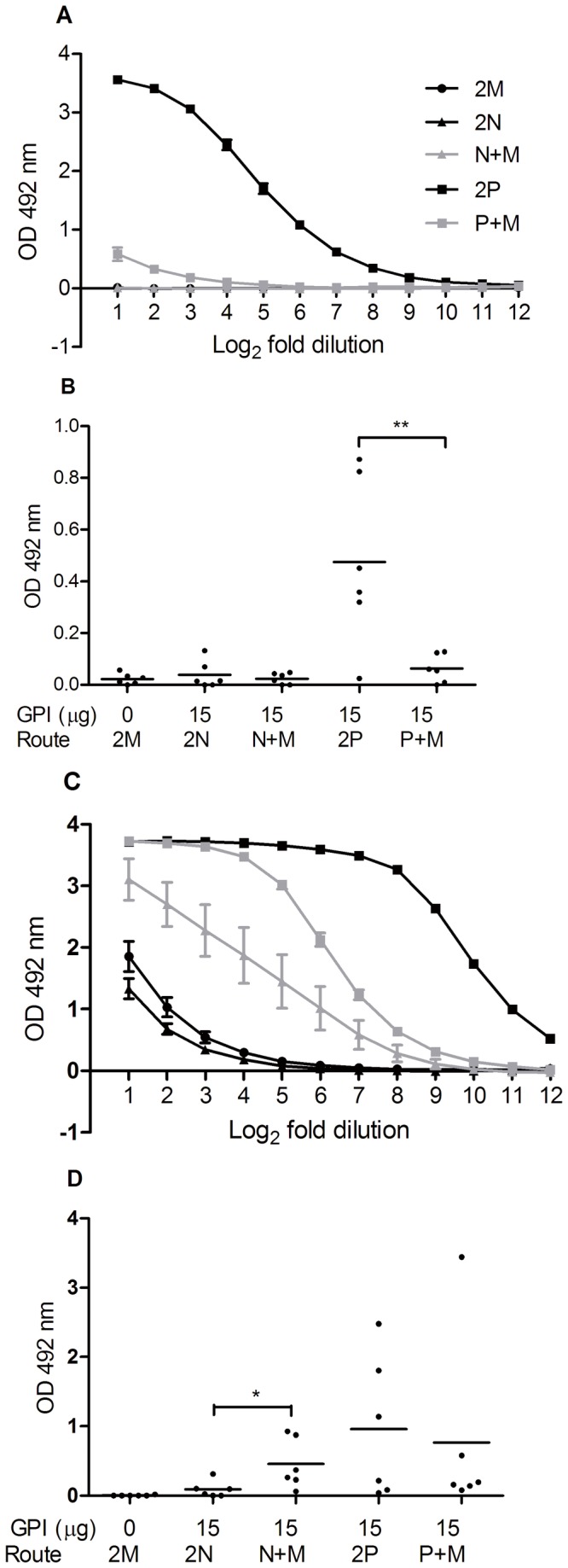
H1N1-specific mucosal SIgA and IgG responses elicited by different immunization strategies. Mice (n=12 per group) were immunized twice with a 20 day interval according to Table 1 and either sacrificed one week later (A, C) or challenged on day 34 and sacrificed 3 days later (B, D). (A) Evaluation of lung SIgA responses after two immunizations. Average OD 492 at each dilution ± standard error of the mean (S.E.M.), n = 6. The starting and ending dilutions are 2 and 4096 respectively. (B) Nasal SIgA responses after two immunizations and live virus challenge. OD 492 readings at 2-fold dilution are given for individual mice. The black line presents the geometric mean per group. (C) Lung IgG responses after two immunizations. Average OD 492 at each dilution ± standard error of the mean (S.E.M.), n = 6 for 2M, 2N and N+M, and n=5 for the other groups. The starting and ending dilutions are 2 and 4096 respectively. (D) Nasal IgG responses after two immunizations and virus challenge. Individual OD 492 at 2-fold dilution with geometric mean per group is given. Groups are named as outlined in Table 1. Levels of significance are depicted as follows: *p < 0.05, **p < 0.01 and ***p < 0.005.

H1N1-specific SIgA ELISA performed on the lung wash samples showed that mice primed with the adjuvanted vaccine via the IN route did not develop detectable lung SIgA titers after mucosal or systemic booster (Figure 1A). In contrast, lung SIgA was readily detectable in mice primed via the IPL route and boosted either via the same route or via the IM route. Yet, the IPL/IPL approach (2P) resulted in significantly higher lung SIgA titers than the IPL/IM approach (P+M; *p*=0.0109). As expected the IM/IM immunized reference group did not develop detectable lung SIgA titers.

H1N1-specific SIgA ELISA on nose wash samples revealed that IN-primed mice did not develop detectable nasal SIgA responses even after a booster via the mucosal or systemic route and challenge with live virus (Figure 1B). In contrast, IPL-primed mice developed robust nasal SIgA responses, but only when the booster was given also via the IPL route (*p*=0.0057 for the comparison between 2P and P+M). Marginal SIgA amounts were found in nose washes from IM/IM-immunized animals.

As mucosal IgG has been suggested to play a role in lung protection against influenza virus infection, we further evaluated the effect of the different immunization strategies on lung and nose IgG responses [9]. Mice primed with the IN vaccine and boosted IM developed significantly higher levels of lung IgG (Figure 1C) and nose IgG (Figure 1D) than mice primed and boosted via the IN route (*p*=0.0474 and 0.02 for the comparison between the 2N and N+M groups in Figure 1C and 1D, respectively). Interestingly, mice primed with the IPL vaccine and boosted IM developed significantly lower lung IgG titers than those immunized IPL during prime and boost (*p*<0.0001 for the comparison between the 2P and P+M group in Figure 1C). With respect to IN IgG titers, there was no difference between these immunization groups. For the IM/IM control group, low levels of IgG were detected in lung washes but not in nose washes.

Taken together, these results indicate that while IPL immunization optimally primed and boosted mucosal antibody responses, IN route was ineffective in priming and boosting mucosal responses against GPI-0100-adjuvanted subunit vaccine.

### Effect of immunization strategy on systemic antibody responses

**Figure 2:**
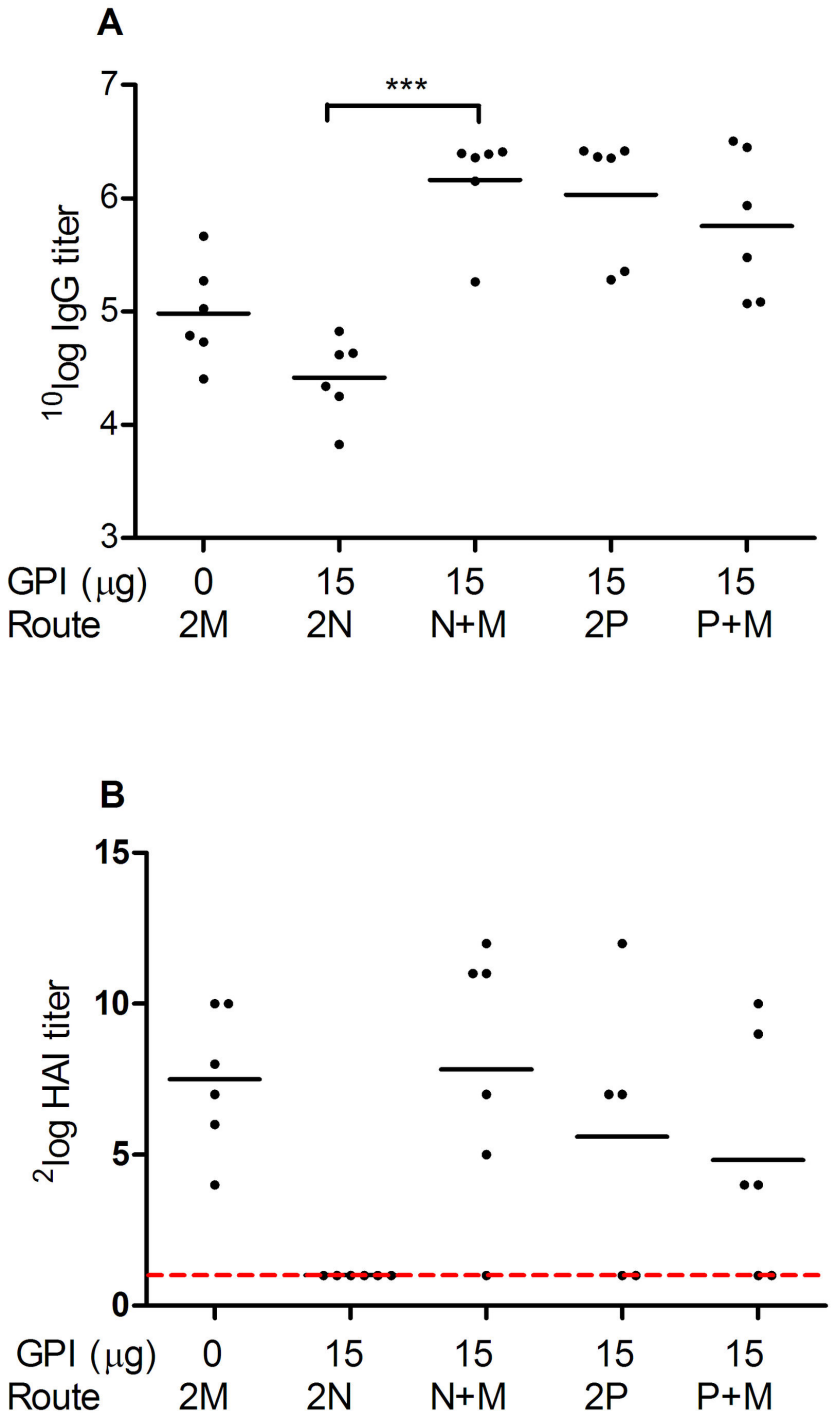
H1N1-specific systemic IgG and hemagluttination inhibition (HAI) titers elicited by different immunization strategies. Serum samples from the mice described in the legend to Figure 1 were collected on day 34 prior to virus challenge (A) and day 37 upon sacrifice (B). (A) Total IgG responses after two immunizations. 10log IgG titers of individual mice and the geometric mean per group are given. (B) HAI titers after two immunizations and virus challenge. Individual 2log HAI titers and the geometric mean per group are given. Due to technical reasons only 5 samples from the 2P treatment group were available for the HAI assay.

We next evaluated the effect of immunization strategy on systemic antibody responses elicited by GPI-0100-adjuvanted influenza vaccine. Both IN, and IPL priming resulted in detectable IgG responses by day 21 (Figure S1). Yet, the titers were significantly lower for IN-primed mice (p=0.0005). Sera of the immunized mice were again collected two weeks after the booster prior to virus challenge. H1N1-specific IgG ELISAs performed on the serum samples showed that mice that received the adjuvanted influenza vaccine by IN/IN immunization (2N) developed an average IgG titer of 4.42, similar to 4.98 from the IM/IM-immunized control group (Figure 2A). The IgG responses in mice immunized by the IN/IM approach (N+M) were significantly enhanced to an average titer of 6.16 (*p*<0.0001 for the comparison between the 2N and N+M group). On the other hand, for IPL-primed mice the booster route had no significant effect on serum IgG responses which were 6.03 and 5.75 for IPL/IPL and IPL/IM-immunized mice, respectively.

Sera of the immunized and challenged mice were collected three days after the challenge upon sacrifice for the evaluation of hemagglutination-inhibition (HAI) titers. Mice immunized with plain influenza vaccine by IM/IM immunization developed an average^2^ logHAI titer of 7.5 (Figure 2B). None of the mice receiving GPI-0100-adjuvanted influenza vaccine by IN/IN immunization developed detectable HAI titers. However, 5 out of the 6 mice receiving the adjuvanted vaccine by the IN/IM approach developed detectable serum HAI titers with an average titer of 7.83, similar to the IM/IM control group. Mice receiving the adjuvanted vaccine IPL/IPL or IPL/IM developed comparable serum HAI titers. With an average titer of 5.6 and 4.8 these were somewhat lower than those obtained by the control group and the IM/IN regimen. Yet, these differences did not reach statistical significance.

Taken together, an immunization strategy involving a mucosal prime followed by a systemic boost improved the systemic antibody responses elicited by IN, but not by IPL vaccine administration.

### Effect of immunization strategy on the phenotype of the immune response

**Figure 3:**
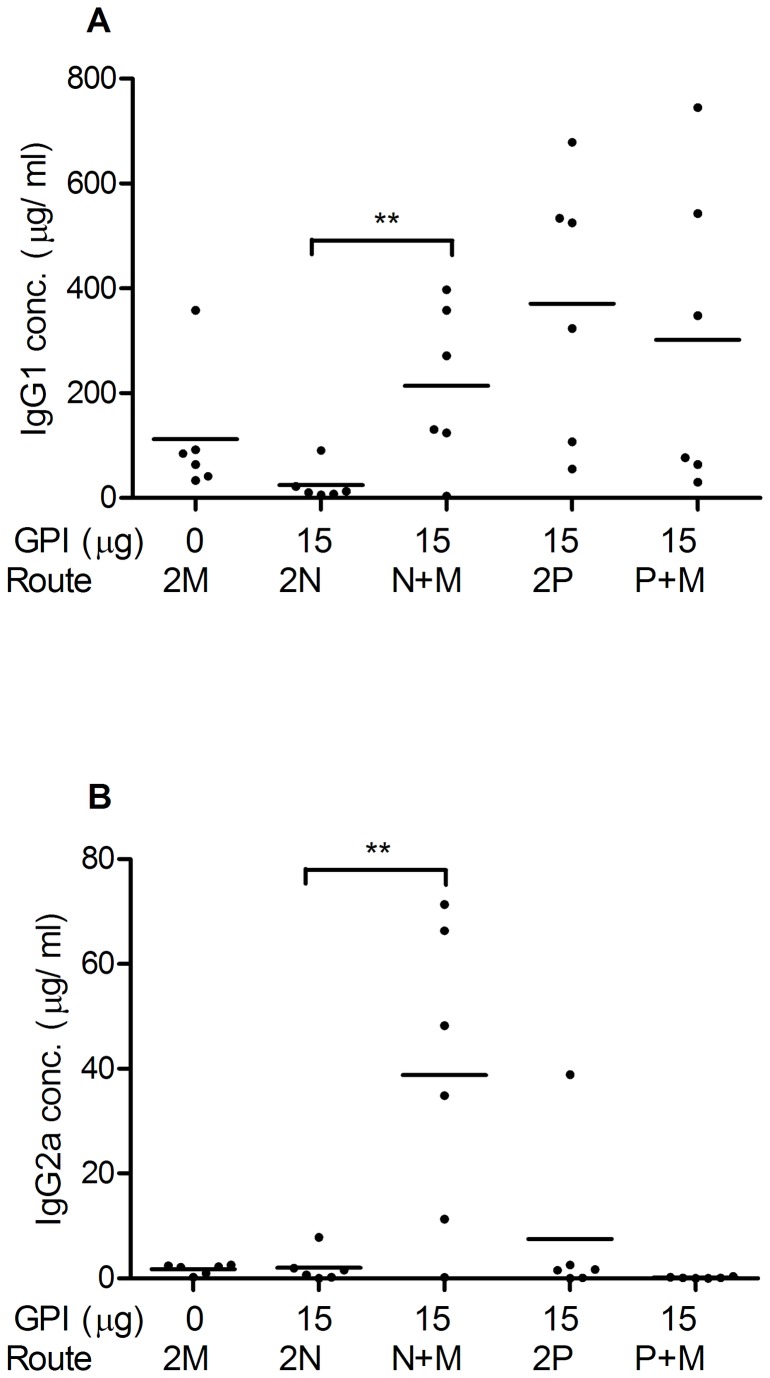
Phenotype of the H1N1-specific systemic antibody responses. Post-challenge serum samples from the mice described in the legend to Figure 1 were analyzed. Individual H1N1-specific IgG1 (A) and IgG2a (B) responses (μg/ml) and the arithmetic means per group are given.

We next examined the phenotype of the antibody responses by performing H1N1-specific IgG1 and IgG2a ELISA assays on the post-challenge serum samples mentioned above. Mice receiving plain influenza vaccine by IM/IM immunization developed serum IgG1 with an average of 113 µg/ml (Figure 3A). Those receiving GPI-0100-adjuvanted influenza vaccine by IN/IN vaccination, however, barely developed detectable serum IgG1 responses. IN/IM delivery of the adjuvanted vaccine resulted in significantly enhanced IgG1 responses with an average of 215 µg/ml (*p*=0.0071). Robust serum IgG1 responses were observed in mice receiving the adjuvanted vaccine by both IPL/IPL and IPL/IM immunization, with an average serum IgG1 of 371 and 301 µg/ml, respectively.

IgG2a responses were low in all mice immunized twice IM, IN, or IPL, except for one mouse in the IPL group (Figure 3B). Interestingly, systemic boost resulted in significantly enhanced serum IgG2a responses in 5 out of the 6 IN-primed mice (*p*=0.0057 for the comparison between 2N and N+M). In contrast, systemic boost had a negative effect if any on IgG2a titers in IPL-primed mice. Irrespective of the immunization strategy, the overall antibody responses elicited by GPI-0100-adjuvanted subunit influenza vaccine were dominated by the Th2-related antibody subtype IgG1.

**Figure 4:**
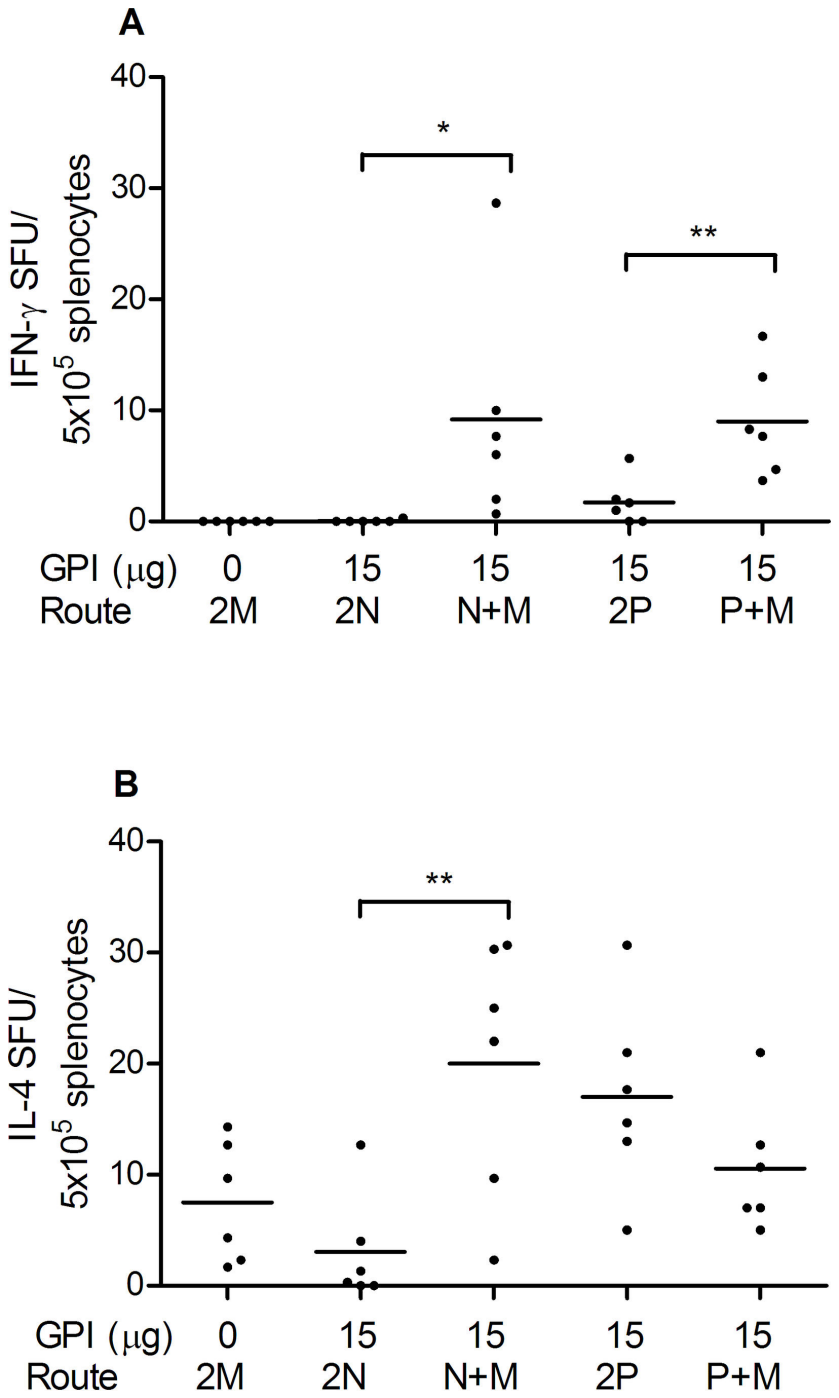
H1N1-specific cellular immunity elicited by different immunization strategies. Post-challenge spleen samples from the mice described in the legend to Figure 1 were collected upon termination. Splenocytes were isolated and stimulated overnight with PR8 subunit. (A) Numbers of H1N1-specific IFN-γ producing cells per 5x10^5^ splenocytes of individual mice are given. (B) Numbers of H1N1-specific IL-4 producing cells per 5x10^5^ splenocytes of individual mice are given. The black line represents the arithmetic mean per group.

To evaluate the effect of immunization strategy on cellular immune responses elicited by GPI-0100-adjuvanted influenza vaccine, spleens of the immunized mice were collected one week after the booster upon termination. H1N1-specific IFN-γ responses were barely detectable in mice receiving plain influenza vaccine IM or those receiving GPI-0100-adjuvanted influenza vaccine IN or IPL (Figure 4A). Interestingly, influenza-specific IFN-γ responses in mucosally-primed mice were significantly enhanced by IM boost as compared to mucosal boost (*p*=0.0264 and 0.004 for the comparison of 2N and N+M and the comparison of 2P and P+M respectively). H1N1-specific IL-4 responses were readily detected in all immunized mice but were relatively lower in mice immunized via the IN/IN regimen. In IN-primed mice, IM boost was significantly more effective then IN boost in enhancing the number of IL-4-producing T cells (p=0.004 for the comparison of 2N and N+M). A similar effect was not observed in IPL-primed mice.

### Effect of immunization strategy on lung protection against influenza virus infection

**Figure 5:**
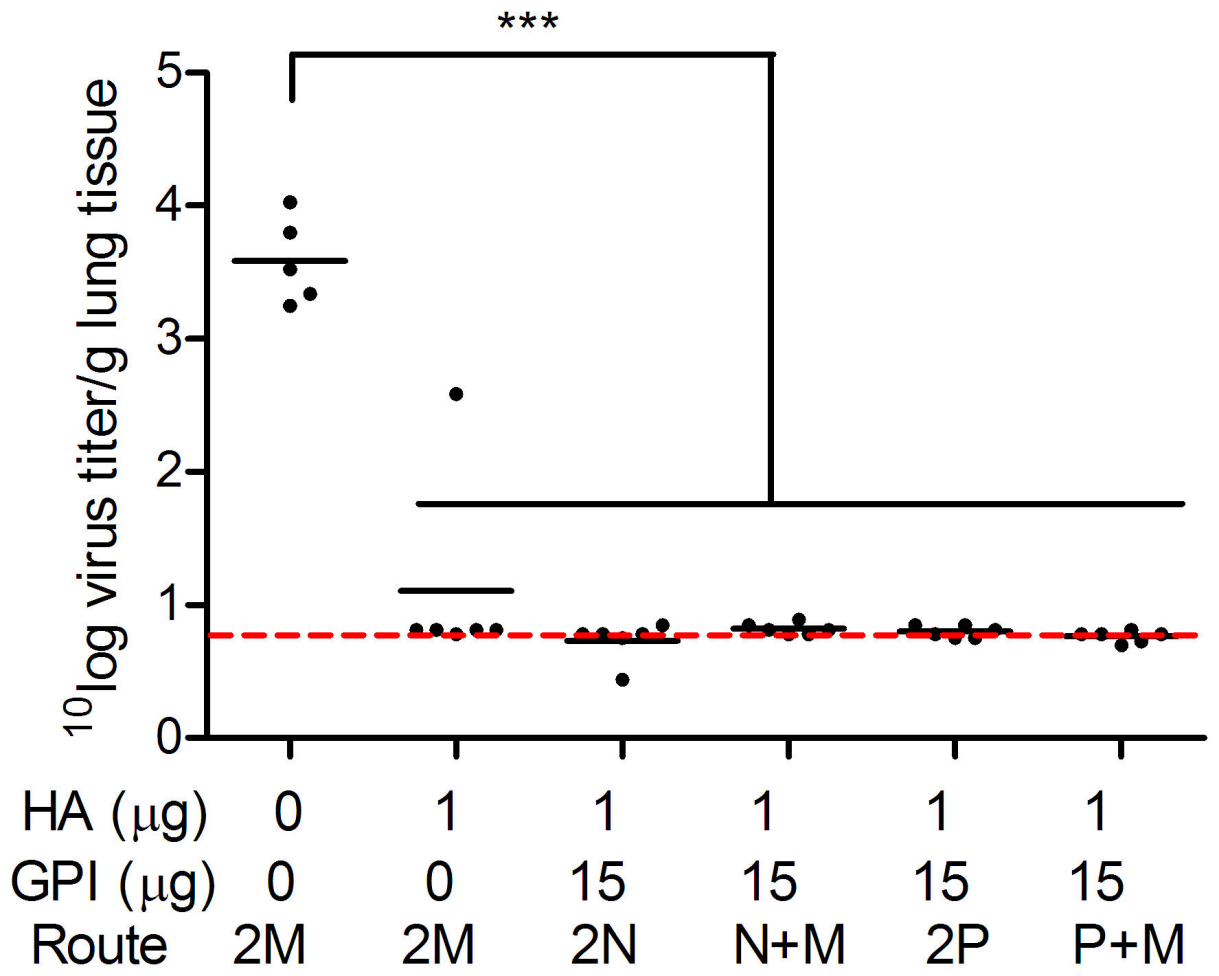
Effect of different immunization strategies on lung virus titers upon challenge. Lung samples from the challenged mice described in the legend to Figure 1 were collected upon termination. Virus titers measured in the lung homogenates are expressed as the 10log virus titer per gram of lung tissue. Individual lung virus titers with the geometric mean titer per group are depicted.

To evaluate the effect of the immunization strategy on protection against virus challenge, the immunized mice were infected with live virus 14 days after the booster immunization and lung virus titers were determined three days later, at the peak of viral replication. Mock-immunized control mice developed an average lung virus titer of 3.59^10^ log/g lung tissue (Figure 5). The lung virus titer was under the detection limit in 5 out of the 6 mice of the IM-immunized control group; one mouse of this group developed a titer of 2.59^10^ log/g lung tissue. Lung virus titers were not detectable in any of the mice that received GPI-0100-adjuvanted influenza vaccine either twice via one of the mucosal routes or by a mucosal prime followed by a systemic boost. Thus, the adjuvanted vaccine provided complete protection of the lungs from virus growth irrespective of the immunization strategy followed.

## Discussion

In the present study, we evaluated the effect of the immunization strategy on the immunogenicity and protective capacity of a GPI-0100-adjuvanted PR8 influenza subunit vaccine. Interestingly, we observed that the optimal boosting route for IN- and IPL-primed mice was different. IN-primed mice developed no mucosal and poor systemic antibody responses when boosted via the IN route. Boosting of IN-primed mice via the IM route, however, resulted in detectable mucosal IgG (though not SIgA) responses, strong systemic antibody responses, enhanced T cell responses and the induction of IgG2a, an antibody subtype associated with improved virus clearance [13]. For IPL-primed mice, IPL/IPL immunization was very effective in inducing mucosal SIgA and IgG as well as systemic antibodies. IPL/IM approach, on the other hand, resulted in relatively modest mucosal antibody responses, although it was equally effective as IPL/IPL strategy for the induction of systemic antibody responses. Despite the different immune profiles, challenge experiments showed that GPI-0100-adjuvanted influenza vaccine delivered by all regimes provided complete protection of the lungs from homologous virus infection.

Our results reveal that mucosal immune responses to prime-boost immunization are affected by both the priming as well as the boosting route. For IN priming, we used a low vaccine volume of 5 µl to retain the antigen in the nasal cavity [31]. This allowed us to clearly distinguish IN from IPL immunization. We observed that IPL but not IN immunization was effective in inducing mucosal SIgA as well as IgG responses. While the robust mucosal antibody titers observed from the IPL/IPL group could be a result of successful induction of local memory responses, the poor mucosal responses in the IN/IN group indicate that IN delivery of influenza vaccine used in the current study barely primed mucosal responses. Yet, IM boost of IN-primed mice though not inducing mucosal SIgA responses did elicit mucosal IgG responses. It has been reported that mucosal IgG can be derived from serum when present in high concentration, and reaches the nose and especially the lungs by transudation [32]. Indeed, except for the IPL/IPL group, mucosal and serum IgG responses elicited from the other groups shared the same trend: IN/IN < IM/IM << IN/IM = IP/IM. The positive correlation between mucosal and serum IgG responses from these groups were confirmed by Spearman analysis (The coefficient for nose IgG vs. serum IgG and lung IgG vs. serum IgG are 0.59 (p=0.0013) and 0.72 (p<0.0001) respectively). Our results are generally in line with an earlier study by Minne and coworkers who tested to which extent the delivery site in the respiratory tract impacts on the immune response elicited by influenza vaccines [33]. These authors used mice primed IN with a whole inactivated virus vaccine administered in a volume of 20 µl. The primed mice were then boosted by administration of split antigen to different parts of the respiratory tract or to the hind muscles. An IN boost resulted in poor mucosal antibody responses (except for nose SIgA) while administration deep into the lungs elicited strong nose and lung SIgA and IgG responses as observed in our study. An IM boost was not effective in stimulating mucosal SIgA, but did boost mucosal IgG responses to similar extents as a boost deep into the lungs. Thus, in agreement with our previous study, we conclude that the IPL route is much more effective than the IN route for priming mucosal antibody responses [28]. Moreover, it is the optimal route for boosting such responses.

Next to mucosal antibodies, systemic IgG antibodies play an important role in protection from severe influenza illness, since they can transudate into the lungs and prevent excessive viral replication and tissue damage upon infection [32]. This is the basis for protection provided by conventional parenterally administered influenza vaccines. In our study, priming via either the IN or the IPL route resulted in detectable serum IgG responses 21 days later, although these responses were significantly lower for IN-primed mice (Figure S1). An IN boost did stimulate the serum IgG responses, but HAI titers could not be detected. An IM boost was essential to achieve systemic antibody responses comparable to or even better than those of the control group immunized IM with non-adjuvanted vaccine. Therefore, even though IN priming elicited only very modest serum antibody responses, memory B cells formed through the priming could be readily activated upon IN or IM boosting. Yet, optimal boosting required antigen administration via the IM route. Our results confirm earlier results from studies on influenza, HSV, HIV-1, and SARS, which all found that IN priming, whether given by protein, DNA or recombinant virus vaccines, should be followed by IM boost for induction of optimal serum antibody responses [21,23,24,33]. For IPL-primed mice, IM and IPL boosting were equally effective in eliciting serum IgG and HAI titers. Also in our earlier study and in the study by Minne et al., an IPL boost was found to result in particularly strong systemic antibody responses [28,33]. Thus, strong systemic antibody responses can be achieved by either two IPL immunizations or IPL priming followed by IM boosting.

In addition to the magnitude, the phenotype of an immune response also determines the effectiveness of its protection against invading pathogens. A Th1 type immune response, characterized by IgG2a and IFN-γ production in mice, has been shown to correlate positively with improved protection against influenza virus [13,30]. Yet, Th1 immunity was barely induced by IM/IM immunization with plain influenza vaccine or IN/IN immunization with GPI-0100-adjuvanted influenza vaccine. IM boosting of IN-primed mice significantly enhanced the Th1 arm of the immune response. The superior quality of the immune responses elicited by IN/IM immunization over IN/IN immunization, and even IM/IM immunization with unadjuvanted vaccine, was in line with earlier studies [21,23,24,33]. As for IPL immunization, GPI-0100-adjuvanted influenza vaccine elicited marginal IgG2a and IFN-γ responses using IPL/IPL approach. Interestingly, IM boosting somewhat decreased the IgG2a response, but significantly enhanced the IFN-γ response of IPL-primed mice. This is in contrast to the study by Minne et al., which showed that IM and IPL boost are both effective in eliciting IgG2a and IFN-γ responses [33]. The different results from the two studies are possibly due to differences in the vaccine formulations used. Minne et al. used a high dose (5 µg HA) of whole inactivated virus (WIV) and a low dose (1.5 µg HA) of split virus for the priming and boosting respectively. WIV possesses natural adjuvant activity from ssRNA (as TLR-7 ligand) and effectively induces Th1 responses [34]. Subunit vaccine used in the current study, on the other hand, is rather ineffective in eliciting Th1 responses and results in a Th2-dominated immune phenotype. Although IM boosting enhances Th1 immunity of IN and IPL vaccines to a different extent, the overall immune responses elicited by GPI-0100-adjuvanted influenza vaccine administered following different immunization strategies were dominated by a Th2 phenotype.

Taken together, immunization strategies involving a mucosal prime followed by a systemic booster or IPL/IPL with properly adjuvanted influenza vaccines are at least as effective as conventional parenteral immunization in inducing systemic antibody responses. This is important since regulatory authorities request that influenza vaccines fulfill quality criteria based on serum HAI titers [35]. Meanwhile, pulmonary immunization probably also raises local memory B cell and T cell responses in the respiratory tract, a phenomenon observed upon influenza infection but not upon intramuscular immunization [36,37]. Thus, mucosal priming is essential for the localization of memory immunocytes to the respiratory tract, which would allow them to respond rapidly to an influenza virus challenge [36,38–40]. Moreover, memory B cells primed by the mucosal, but not the systemic, route preferentially express SIgA, which is the major antibody subtype which mediates early immune exclusion and also exhibits cross-protective capacity. Hence, IN/IM, IPL/IM or IPL/IPL immunization regimens should be further explored to come to optimized immunization regimens for protection from respiratory viral infections.

## Supporting Information

Figure S1 H1N1-specific systemic IgG primed by different immunization routes. Serum samples from the mice described in the legend to Figure 1 were collected on day 20 prior to the second immunization. Total IgG responses from 2M, N+M and P+M groups after priming are given.
(TIF)
