# Supplementary figures and images for: Evaluation of Mucosal and Systemic Immune Responses Elicited by GPI-0100- Adjuvanted Influenza Vaccine Delivered by Different Immunization Strategies
Source: PLoS One. 2013 Jul 31;8(7):e69649. doi: 10.1371/journal.pone.0069649 (PMC3729563; doi:10.1371/journal.pone.0069649)

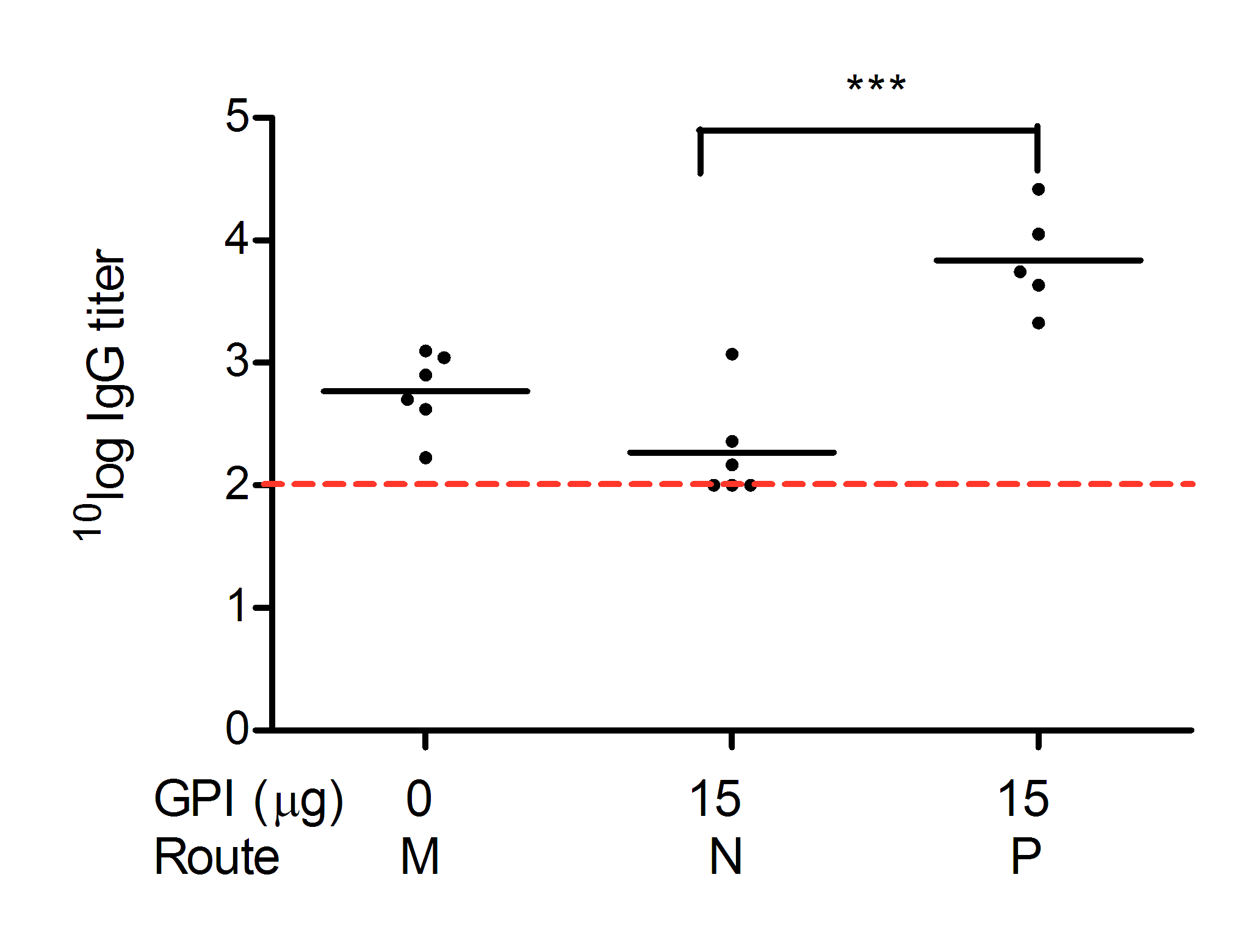

Supplement: Figure S1 — Serum samples from the mice described in the legend to Figure 1 were collected on day 20 prior to the second immunization. Total IgG responses from 2M, N+M and P+M groups after priming are given. (TIF) [file pone.0069649.s001.tif]
